# The Species-Specific Inversion Polymorphism of the X Chromosome in *Anopheles messeae* and *Anopheles daciae* Is Based on the Common Ancestral Variant X1

**DOI:** 10.3390/genes17010005

**Published:** 2025-12-19

**Authors:** Evgeniya S. Soboleva, Maria V. Sharakhova, Igor V. Sharakhov, Gleb N. Artemov

**Affiliations:** 1 Laboratory of Evolutional Cytogenetics, Tomsk State University, 36 Lenin Avenue, Tomsk 634050, Russia; jane.sable.me@gmail.com (E.S.S.);; 2 Laboratory of Cell Differentiation Mechanisms, Institute of Cytology and Genetics, 10 Lavrentyev Prospekt, Novosibirsk 630090, Russia; 3 Department of Entomology, The Fralin Life Sciences Institute, Virginia Polytechnic Institute and State University, 360 West Campus Drive, Blacksburg, VA 24061, USA

**Keywords:** *Anopheles*, malaria mosquitoes, inversion polymorphism, X chromosome, chromosomal rearrangements, inversion, evolution

## Abstract

**Background/Objectives**: Chromosomal inversions play an important role in the evolution of insects by forming genetic barriers between closely related species and facilitating local adaptation. Polymorphic inversions in malaria mosquitoes of the Maculipennis subgroup have been studied for over 50 years, yet the evolutionary ancestry of the gene orders remains unknown. In this study, we mapped the genes flanking the breakpoints of two polymorphic X-chromosome inversions in the cryptic species *Anopheles messeae* and *Anopheles daciae* of the Maculipennis subgroup. **Methods**: We used an iterative mapping approach to define the breakpoint regions, selecting flanking markers based on the genome assembly of the reference species, *Anopheles atroparvus*. To identify the ancestral X chromosomal arrangement in *An. messeae* and *An. daciae*, we developed and implemented the genomic inversion calculator (GIC), which uses greedy heuristics to determine the shortest evolutionary scenario of rearrangements. **Results**: Our knowledge of the relative genomic positions of the inversion breakpoints in *An. daciae* and *An. messeae* enabled us to use the *An. atroparvus* genome as an outgroup and the GIC tool to show that the X0 and X2 arrangements emerged independently along the evolutionary lineages of *An. daciae* and *An. messeae*, respectively, based on the X1 arrangement. **Conclusions**: These results refine the structure and boundaries of the X chromosome rearrangements and reconstruct the sequence of evolutionary events in the cryptic complex *An. messeae*–*An. daciae*, demonstrating that the X1 arrangement is ancestral. This study lays the groundwork for analyzing the molecular organization of breakpoints, the mechanisms of inversion formation, and their role in speciation.

## 1. Introduction

Chromosomal inversions are a type of structural genomic rearrangement, in which a segment of a chromosome flips 180 degrees, resulting in a reversal of the genetic material’s order. Inversions have been observed to accompany the evolution and adaptation of dipteran insects, including mosquitoes [1,2]. Inversions can cause a reduction in genetic recombination and altered expression of genes in the vicinity of breakpoints [3,4]. Mosquitoes of the genus *Anopheles* can transmit malaria, which kills more than 600,000 people per year [5]. *Anopheles* mosquitoes are also vectors of filariasis and viral infections, causing significant economic damage and reducing the quality of life of the human population [6,7].

The frequency of chromosomal inversions in natural populations of malaria mosquitoes frequently exhibits a non-random geographical distribution, correlating with climatic conditions such as temperature and humidity. This suggests their involvement in adaptation to local environmental conditions [8,9,10,11,12,13]. Furthermore, some inversions have been demonstrated to be associated with insecticide resistance [2,14] and feeding behavior [15,16], which are traits of significant epidemiological importance [17]. A comparative genomics study has demonstrated that the X chromosome in malaria mosquitoes has accumulated fixed inversions at a rate that is three times faster than autosomes [18]. Such elevated levels of genetic variation may indicate a central role of the X chromosome in the formation of reproductive isolation and speciation.

Two mosquito species, *Anopheles messeae* Falleroni, 1926 and the more recently described *An. daciae* Linton, Nicolescu & Harbach, 2004 [19], belong to the Palearctic Maculipennis subgroup. These species exhibit pronounced inversion polymorphisms compared to other species in the subgroup [20]. At least two polymorphic variants have been described and mapped for each chromosome of *An. messeae* and *An. daciae*. The remaining species of the subgroup are partially or completely chromosomally monomorphic [8]. *Anopheles messeae* and *An. daciae* are distributed in the temperate latitudes of Eurasia and, in conjunction with *An. beklemishevi* Stegnii & Kabanova, pose a potential threat to public health because, in addition to malaria, they can carry parasitic nematodes [21,22,23]. *Anopheles daciae* is a cryptic species that was distinguished from *An. messeae s.l.* based on nucleotide substitutions in the sequences of the internal transcribed spacer of ribosomal DNA (ITS2) [19,24]. The evolution of the X chromosome in *An. messeae* and *An. daciae* was associated with two nested paracentric inversions based on the ancestral gene order present in *An. atroparvus* and *An. maculipennis* [25]. The two nested inversions were fixed in both *An. messeae* and *An. daciae*, giving rise to the X1 arrangement.

The standard chromosomal arrangements in the X chromosomes of the Maculipennis subgroup have traditionally been denoted as 00 in a homozygote and 0 in a hemizygote. Inverted arrangements are denoted as 11, 22, 33, etc., in a homozygote and 1, 2, 3, etc., in a hemizygote [8]. Standard chromosomal arrangements have been assumed to be ancestral in the Maculipennis subgroup. However, a robust analysis of the chromosomal arrangement’s ancestry based on outgroups has not yet been performed. A new standard universal cytogenetic map was recently developed for *An. messeae* and *An. daciae*. This map is based on the X11, 2R00, 2L00, 3R00, and 3L00 karyotype [26]. The X11 karyotype was used because a recent study indicated that the X00 karyotype is either extremely rare or absent in *An. messeae* populations [27].

Polymorphic inversions 3R1 and 3L1 are shared by *An. messeae* and *An. daciae*, whereas 2R and X chromosomal inversions are species-specific. *Anopheles messeae* is characterized by the X12 polymorphism (X1 and/or X2 arrangements), as well as the 2R01 polymorphism, whereas *Anopheles daciae* is characterized by the X01 polymorphism (X0 and/or X1 arrangements) [20,26,27]. Although fixed chromosomal rearrangements in the X chromosome are known to occur frequently, polymorphic inversions in the sex chromosome are rare in malaria mosquitoes [28,29]. In the Maculipennis subgroup, polymorphic inversions in the X chromosome occur in only three species: *An. messeae*, *An. daciae*, and *An. beklemishevi*. Polymorphic inversions can be a potential causative agent in the process of speciation [30]. However, inversion polymorphism has also been shown to be a product of natural selection, with the capacity for prolonged persistence within natural populations [31].

The purpose of this study was to reconstruct the structural evolution of the X chromosome in the *An. messeae* and *An. daciae* phylogenetic branch by determining the breakpoint regions of the polymorphic inversions and comparing their location with that in the genome of the outgroup species.

## 2. Materials and Methods

### 2.1. Biological Material

The material for the study was fourth-instar larvae of *Anopheles* mosquitoes collected from natural populations in the Tomsk region (Russia) during the summer seasons of 2018–2021. The larvae were fixed in Carnoy’s fixative (96% ethanol–acetic acid, 3:1) and stored at −20 °C.

### 2.2. Air-Dried Preparations of Polytene Chromosomes

Preparations of polytene chromosomes from salivary gland cells were prepared using a standard method, freezing the prepared pressed unstained preparations in liquid nitrogen to remove the cover glass, followed by dehydration in ethanol [32].

### 2.3. Species Identification

Identification of *An. messeae* and *An. daciae* individuals was performed using PCR-based Restriction Fragment Length Polymorphism (PCR-PCRF) analysis by comparing the lengths of the ITS2 region sequences [26]. Species-specific nucleotide substitutions at restriction sites allow for the digestion and undigestion of PCR products, resulting in length-based species diagnostics.

### 2.4. Genomic and Physical Mapping of Inversion Breakpoints

To determine the location of inversion breakpoints on chromosome X, an iterative mapping method was used [25]. This method was based on the physical mapping of genetic markers located in the vicinity of the regions containing them. In this study, the exons of the reference assembly *An. atroparvus* AatrE3 [18,33] on both sides of the presumed breakpoint were used as markers. The choice of marker in each repetition of the experiment (iteration) depended on the results of the previous one, namely, whether the marker was mapped inside or outside the rearrangement. The result of iterative mapping was a region containing the breakpoint of the rearrangement enclosed between the exons of genes or within a single gene of the reference genome. DNA markers developed based on the AatrE3 assembly were designated with six-digit IDs corresponding to annotated genes. Large gene exons (at least 500 bp in length) were used as probes. If a gene’s exons were 100–200 bp long, then two exons and the intron between them were used as a probe. The probes were mapped using the banding pattern of polytene chromosomes stained with DAPI.

### 2.5. Reconstruction of Genomic Rearrangements

To reconstruct genomic rearrangements, we used our proprietary software tool, Genome Inversions Calculator (GIC), which implements an algorithmic solution to minimize the number of reversals (inversions) when converting one order of syntenic blocks to another within a single chromosome. This problem is known in combinatorial bioinformatics as the “reversal distance problem” [34]. The conceptual implementation of GIC was based on the GRIMM (Genome Rearrangements in Man and Mouse) tool [35]. The output includes a clear visual representation of the inversion process and a minimal number of rearrangements required to transform one syntenic order into another (Figure S1). Therefore, GIC fills the methodological gap between fully automated genome rearrangement systems and manual comparative analyses, offering an intuitive interface for exploring the logic of chromosomal evolution in specific genomic contexts.

The tool is distributed under the Apache 2.0 license and is available in an open repository: https://github.com/janesable/gic.git (accessed on 17 September 2025).

## 3. Results

### 3.1. Breakpoint Regions of the Polymorphic Inversions on the X Chromosome in An. daciae and An. messeae

Previous studies have indicated that the X1 arrangement is shared by *Anopheles messeae* and *An. daciae*, while the X0 arrangement is specific to *An. daciae* and the X2 arrangement is specific to *An. messeae* [20,26,27]. Thus, we hypothesized that both the X0 and X2 arrangements originated from the X1 arrangement. To test this hypothesis, we mapped the breakpoint regions of these three arrangements using exons from the chromosome-level genome assembly of *An. atroparvus* [18,33]. Regions containing breakpoints of polymorphic inversions were physically mapped on the cytogenetic map of the salivary glands of *An. messeae* [26] and the genomic map of *An. atroparvus* [18,33]. For the FISH mapping of breakpoint regions, 22 genetic markers (Table S1) from a previously developed set of 53 markers were used for the analysis of the fixed rearrangements of the X chromosome [25].

The region containing the distal breakpoint of the X2 arrangement in *An. messeae* was mapped with a resolution of 26.6 Kbp, while the region containing the proximal breakpoint was mapped with a resolution of 14.8 Kbp. For the X0 arrangement of *An. daciae*, the proximal breakpoint was mapped with a resolution of 8.5 Kbp, while the length of the region containing the distal breakpoint was 171.5 Kbp (Table 1).

**Figure 1:**
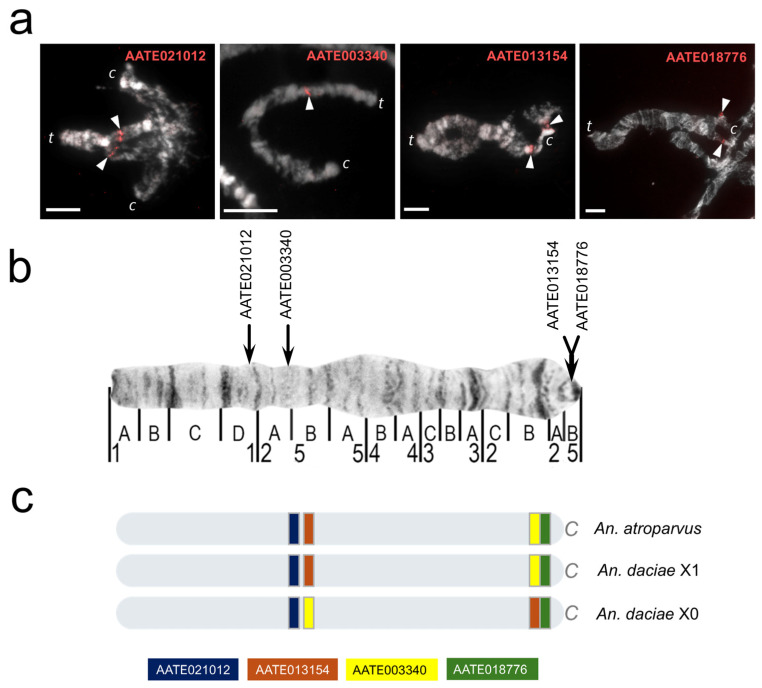
Location of DNA markers flanking breakpoint regions of the X chromosome in *An. atroparvus* and *An. daciae*. (**a**) Localization of DNA markers on polytene chromosomes of salivary gland cells of *An. daciae* individuals with genotype X00 (and X01 for AATE021012). (**b**) Physical map of DNA markers on chromosome X00. (**c**) Comparison of the position of DNA markers relative to the reference genome of *An. atroparvus*, as well as the order of genes in X1 and X0 in *An. daciae*. c—centromere, t—telomere, white arrowheads—FISH signals, and scale bar = 10 μm.

The region, which spans 171.5 Kbp and contains the distal breakpoint of the polymorphic inversion X1, contains only 10 annotated genes. These could not be mapped to the X chromosome of *An. daciae* (see Figure 1). The ortholog of gene AATE013223 was localized in the 3R arm of *An. daciae*, while for other orthologs (AATE009672, AATE006112), the developed DNA probes did not hybridize on the chromosomes of *An. daciae*. The remaining seven genes contained only short exons (no longer than 200 bp), which made it difficult to develop DNA probes for them. Furthermore, some of these genes contained nonunique sequences in the genome.

**Figure 2:**
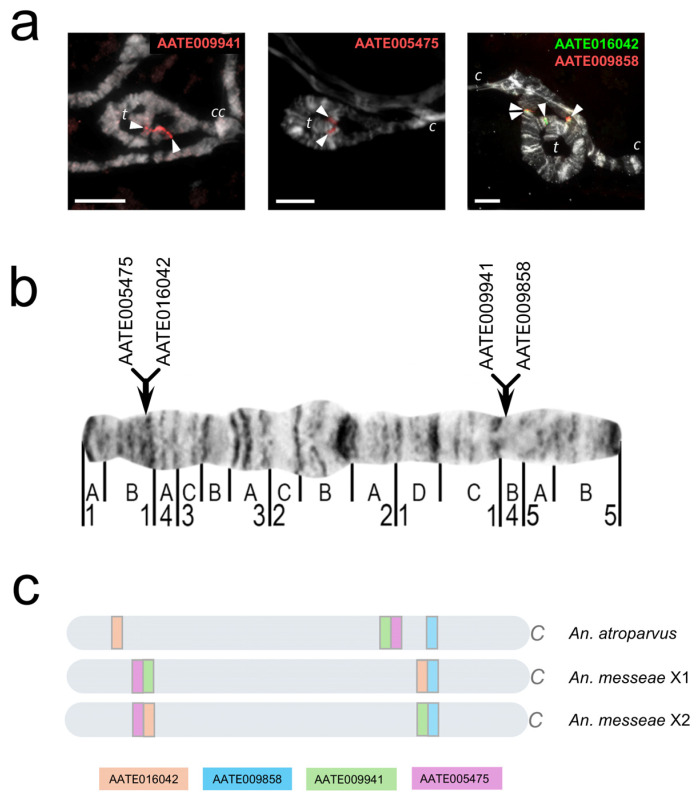
Location of DNA markers flanking breakpoint regions of the X chromosome in *An. atroparvus* and *An. messeae*. (**a**) Localization of DNA markers on polytene chromosomes of salivary gland cells of *An. messeae* individuals with the X12 genotype. (**b**) Physical map of DNA markers on chromosome X22. (**c**) Comparison of the position of DNA markers relative to the reference genome of *An. atroparvus*, as well as the order of genes in X1 and X2 in *An. messeae*. c—centromere, cc—chromocenter, t—telomere, white arrowheads—FISH signals, and scale bar = 10 μm.

The mapping of the distal breakpoint of the X2 inversion revealed its location within the interval between the breakpoints of two previously described fixed inversions [25]. The region containing the proximal breakpoint of the X2 inversion is entirely congruent with the breakpoint region IV (BRIV) of the fixed inversion, where the same genes AATE016042 and AATE009858 were utilized as markers (see Figure 2). Physical mapping of genetic markers on the cytogenetic map of polytene chromosomes of salivary gland cells confirmed the location of the breakpoints of inversion X2 in regions 1B–C and 4B, and the proximal breakpoint of inversion X1 in region 5B [26]. The accuracy of determining the position of the distal breakpoint of the X1 inversion was lower than that of the cytogenetic breakpoint (1D–2A).

### 3.2. The Ancestral X Chromosome Arrangement in An. messeae and An. daciae

**Figure 3:**
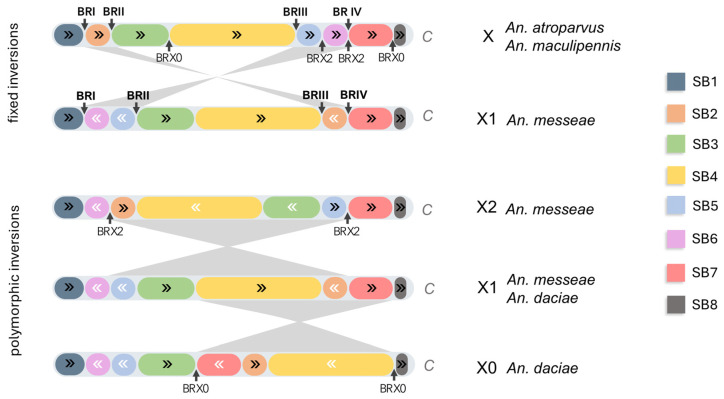
The order and orientation of syntenic blocks are formed by breakpoints of fixed and polymorphic inversions in *An. messeae* and *An. daciae*. C—centromeric end of the chromosome; BR—breakpoint region; SB—syntenic block; and arrows indicate changes in the order of genes within the syntenic blocks.

Here we explore the ancestry of the X chromosomal arrangements in *An. messeae* and *An. daciae*. The presence of the X1 arrangement in the *An. messeae* and *An. daciae* lineages is the result of two nested paracentric inversions based on the X ancestral chromosome that are present in *An. atroparvus* and *An. maculipennis* [25]. The breakpoint regions of the two fixed inversions—‘outer’ (BRI and BRIV) and ‘inner’ (BRII and BRIII)—divide the X chromosome into five syntenic blocks. It is intriguing to test whether the polymorphic inversions in *An. messeae* and *An. daciae* disrupt these five syntenic blocks. Our mapping shows that the breakpoints of the X01 inversion polymorphism in *An. daciae* disrupts two of five syntenic blocks, while the X12 inversion polymorphism in *An. messeae* disrupts only one of five syntenic blocks, since the proximal breakpoint region of the X2 arrangement colocalizes with the proximal BRIV of the ‘outer’ fixed inversion (Figure 3). Therefore, when breakpoint regions of both fixed and polymorphic inversions are mapped to the X chromosome, the ancestral arrangement is divided into eight syntenic blocks (SB1–SB8).

The length of syntenic blocks ranged from 0.5 Mbp (SB2) to 7.7 Mbp (SB4) based on the *An. atroparvus* AatrE3 genome. The length of SB8 cannot be accurately estimated, since the location of the centromeric region in the *An. atroparvus* genome is not defined. However, the improved assembly of the AatrE4 genome [36] allows the estimation of SB8 to be at least 8.5 Mbp. It is evident that SB1, SB3, SB4, SB7, and SB8 in the X1 arrangement have maintained the ancestral order, while SB2, SB5, and SB6 have undergone alterations in both their location on the chromosome and the order of genes. The X12 inversion polymorphism involves four syntenic blocks (SB2, SB3, SB4, and SB5), while the X01 polymorphism involves three syntenic blocks (SB2, SB4, and SB7). If the lengths of polymorphic inversions are represented as the sum of the lengths of syntenic blocks in the *An. atroparvus* genome, then the lengths of both inversions are approximately equal. The length of X12 is approximately 11.7 Mbp (45% of the total length of X chromosome), and the length of X01 is 11.2 Mbp (43% of the total length of X).

**Figure 4:**
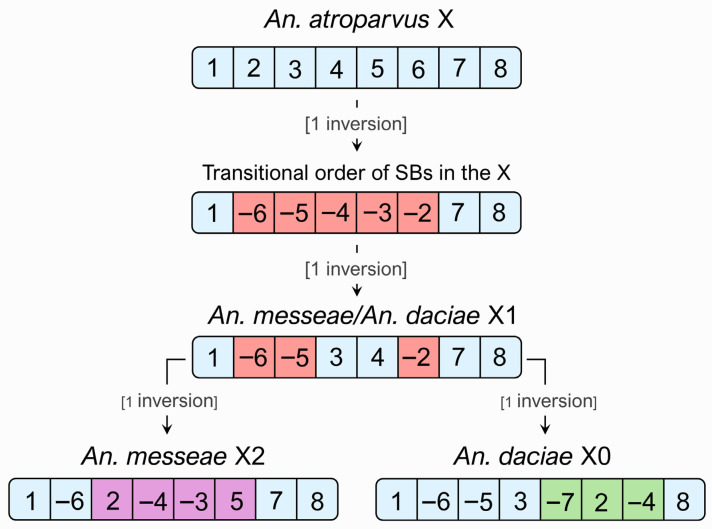
Evolution of gene order in the X chromosome of *An. messeae* and *An. daciae* based on GIC analysis results. Syntenic blocks affected by fixed inversions are marked in red, while polymorphic inversions in *An. messeae* and *An. daciae* are marked in purple and green, respectively. Note: It is equally likely that the two nested inversions between *An. atroparvus* and *An. messeae*/*An. daciae* occurred in the opposite order: 12345678 → 12 −4 −3 5678 → 1 −6 −5 34 −2 78.

To reconstruct the evolutionary history of the X chromosome, we applied the GIC algorithm, which was developed to infer inversion scenarios within chromosomes. Unlike GRIMM, which supports several types of rearrangements (inversions, translocations, fusions, and splits) between different chromosomes, GIC was specifically designed to analyze inversions within a single chromosome. Instead of the Hannenhalli–Pevzner polynomial algorithm implemented in GRIMM, GIC applies a simplified set of greedy heuristics, which reduces computational complexity and provides an intuitive interface. This design makes the tool accessible to researchers without bioinformatics training who work with data from physical mapping, sequencing, or cytogenetic studies. While this simplification restricts the analysis to single-chromosome rearrangements, it provides a transparent framework ideally suited for reconstructing inversion-based chromosomal evolution. Thus, among the one million possible scenarios for the transformation of the ancestral gene order to the orders seen in the *An. messeae* and *An. daciae* lines, the GIC algorithm exhibited a single shortest scenario for each species. Accordingly, the X1 arrangement, which is common to *An. daciae* and *An. messeae*, was shown as a step prior to the formation of the X2 and X0 arrangements in both species, supporting our hypothesis (Figure 4). Therefore, we propose to refer to the X0, which is common in natural populations of *An. daciae*, and X2, which is common in *An. messeae*, as inverted arrangements and to the X1 as an uninverted arrangement.

## 4. Discussion

The high-quality genome assemblies of organisms accumulated to date allow researchers to use comparative genomics approaches to map chromosomal rearrangements in closely related species whose genome sequences have not been developed. The method of iterative mapping of regions containing breakpoints of chromosomal rearrangements used in this study has proven to be applicable to species for which a chromosome-level genome assembly is not available. The results of mapping polymorphic inversions and fixed inversions [25] in the X chromosome of *An. messeae* and *An. daciae* demonstrate the good resolution of the method, which depends mainly on the density of marker genes in the region of interest of the reference genome, although non-coding sequences can also serve as markers, if necessary. During iterative mapping of the entire set of genetic markers surrounding the breakpoints, the position of the breakpoints and the regions containing them are refined. Despite its applicability, the iterative mapping method is inferior to other approaches such as Hi-C [36,37] or nucleotide sequence alignments of the alternative variants of chromosomal arrangements [38,39]. These approaches allow obtaining information about breakpoints with nucleotide accuracy, as well as describing the neighborhoods of breakpoints. Furthermore, iterative mapping is not suitable for organisms for which high-quality polytene chromosome preparations cannot be obtained. This method works best with dipteran insects, for which polytene chromosomes can be used to detect even small (a few Mbs) chromosomal rearrangements. The X12 inversion polymorphism in *An. messeae* involves a central part in the left arm of chromosome X that covers approximately half of its physical length, which roughly corresponds to the location of previously discovered fixed inversions on this chromosome [25]. The distal breakpoint region of the X2 arrangement is located between the breakpoints of fixed inversions, while the proximal breakpoint region of the X2 arrangement coincides with the BRIV of the fixed inversion, which may represent a “fragile region”. As demonstrated in Soboleva et al. [25], there is an increased content of transposable elements and simple repeats in the vicinity of the breakpoints of fixed inversions in the *An. atroparvus* genome. These elements could act as factors initiating inversions by a recombinant mechanism. A similar enrichment of repetitive elements in the vicinity of breakpoints has been demonstrated in various organisms, including *Anopheles* and *Drosophila* [4,38,40]. For instance, the breakpoints of the 2Rb inversion in *An. gambiae* are located in tandem repeat regions several thousand base pairs in length [38]. As demonstrated by studies in *Drosophila*, inversion breakpoints frequently coincide with boundaries of topologically associating domains, which are characterized by structural instability [41,42].

The X01 inversion polymorphism in *An. daciae* also involves a central part in the left arm of chromosome X, but the resolution of the distal breakpoint region mapping was lower. The low mapping resolution in the distal breakpoint region of the X0 arrangement in *An. daciae* could be due to low gene density and high repeat content. If this is the case, this region could represent a “fragile region”, similar to those found in *Drosophila* [43]. The “fragile region” model suggests that chromosomal rearrangements occur more frequently in areas characterized by low gene density and high repeat content. This hypothesis can be confirmed with the availability of a whole-genome assembly for *An. daciae*.

The physical mapping results allowed us to reconstruct the scenario of the X chromosome evolution in the Maculipennis subgroup. Traditionally, the X0 arrangement was considered evolutionarily ancestral, while the X1 and X2 arrangements were considered derived [8]. However, our data demonstrates that the position and orientation of syntenic blocks in the X1 variant represent the ancestral gene order in *An. daciae* and *An. messeae*, while the X0 and X2 inversion variants arose independently on the basis of the X1 arrangement. This conclusion is based on the fact that the X1 variant is only two inversion steps away from the ancestral X chromosome arrangement present in the outgroup species *An. atroparvus* [25]. The X0 and X2 variants require one more inversion step each from the X1 variant. A parallel situation can be seen in the case of the 2La/+a inversion polymorphism in the African mosquito *An. gambiae*. For a long time, the 2L+a arrangement was considered ancestral and the 2La arrangement was considered derived [28,44]. However, the genomic analyses of the inversion breakpoints demonstrated that the opposite is true for the 2La/+a polymorphic inversion, i.e., the 2La arrangement is ancestral [45,46].

The X2 inversion of *An. messeae* is typically observed at a low frequency in natural populations of the West Siberian Plain [8,20,47]. It is hypothesized that the X2 inversion is associated with local adaptations to the habitat of *An. messeae* in the floodplains of the Ob and Irtysh rivers in Western Siberia. The X0 inversion of *An. daciae* is widespread throughout the species’ range, with a frequency of approximately 48%. The emergence of the X0 inversion in *An. daciae* may have contributed to its spread in conditions of reduced humidity and higher summer and average annual temperatures in the steppe and forest-steppe zones of Eurasia. Both polymorphic inversions have been observed in interspecific hybrids distributed in the central and southern parts of the West Siberian Plain [20]. Nevertheless, in all populations of both species, as well as in hybrids, the ancestral X1 arrangement is more common. The high frequency of the X1 variant may be the result of an extensive selection process in the diverse habitats of the two mosquito species.

## 5. Conclusions

The molecular mapping of the breakpoints of polymorphic inversions X2 and X0 in *An. messeae* and *An. daciae* allowed us to reconstruct the evolution of the X chromosome in the Maculipennis subgroup. Our study demonstrated that the most probable scenario is the emergence of polymorphic inversions X0 and X2, based on the X1 gene order variant in *An. daciae* and *An. messeae*, respectively. The results obtained provide a basis for further analysis of chromosomal phylogeny, as well as for studying the role of inversions in the species divergence and adaptation to local habitat conditions.

## Figures and Tables

**Table 1:** The coordinates of the BR-flanking gene markers in the *Anopheles atroparvus* genome assembly.

Breakpoint Region	Distal DNA Marker			Proximal DNA Marker		
	Gene Position, bp	Gene ID	Exons *	Gene Position, bp	Gene ID	Exons *
X2 distal	13,261,242… 13,265,420(+)	AATE009941	2–3	13,236,449… 13,246,292(−)	AATE005475	3
X2 proximal	1,465,925… 1,468,608(−)	AATE016042	2–3	14,520,988… 14,521,746(+)	AATE009858	1
X0 distal	4,398,009… 4,404,032(−)	AATE021012	4	4,571,860… 4,577,721(−)	AATE013154	3
X0 proximal	17,252,047… 17,256,383(+)	AATE003340	2	17,260,494… 17,265,377(−)	AATE018776	2–4

* Exons for which the probes were designed.

## Data Availability

The data and materials used in this study data are archived in a publicly accessible repository at https://doi.org/10.5281/zenodo.17533993 (accessed on 5 November 2025).

## Supplementary Materials

The following supporting information can be downloaded at https://www.mdpi.com/article/10.3390/genes17010005/s1. Figure S1: Illustration of the GIC tool in operation; Table S1: *Anopheles atroparvus* marker genes used to map the breakpoints of the X chromosome polymorphic rearrangements in *An. messeae* and *An. daciae* using the AatrE3 assembly; Table S2: Characteristics of syntenic blocks based on the AatrE3 assembly.

## Abbreviations

The following abbreviations are used in this manuscript:

AatrE3	*Anopheles atroparvus* EBRO genome assembly
BR	Breakpoint region
FISH	Fluorescence in situ hybridization
ITS2	Internal transcribed spacer of ribosomal DNA
PCRF	Restriction fragment length polymorphism
SB	Syntenic block
