# Supplementary material for: The Species-Specific Inversion Polymorphism of the X Chromosome in Anopheles messeae and Anopheles daciae Is Based on the Common Ancestral Variant X1
Source: Genes (Basel). 2025 Dec 19;17(1):5. doi: 10.3390/genes17010005 (PMC12841409; doi:10.3390/genes17010005)
Supplement: Supplementary file 1 [file genes-17-00005-s001.zip › genes-4017398-supplementary.pdf]

Supplementary Materials

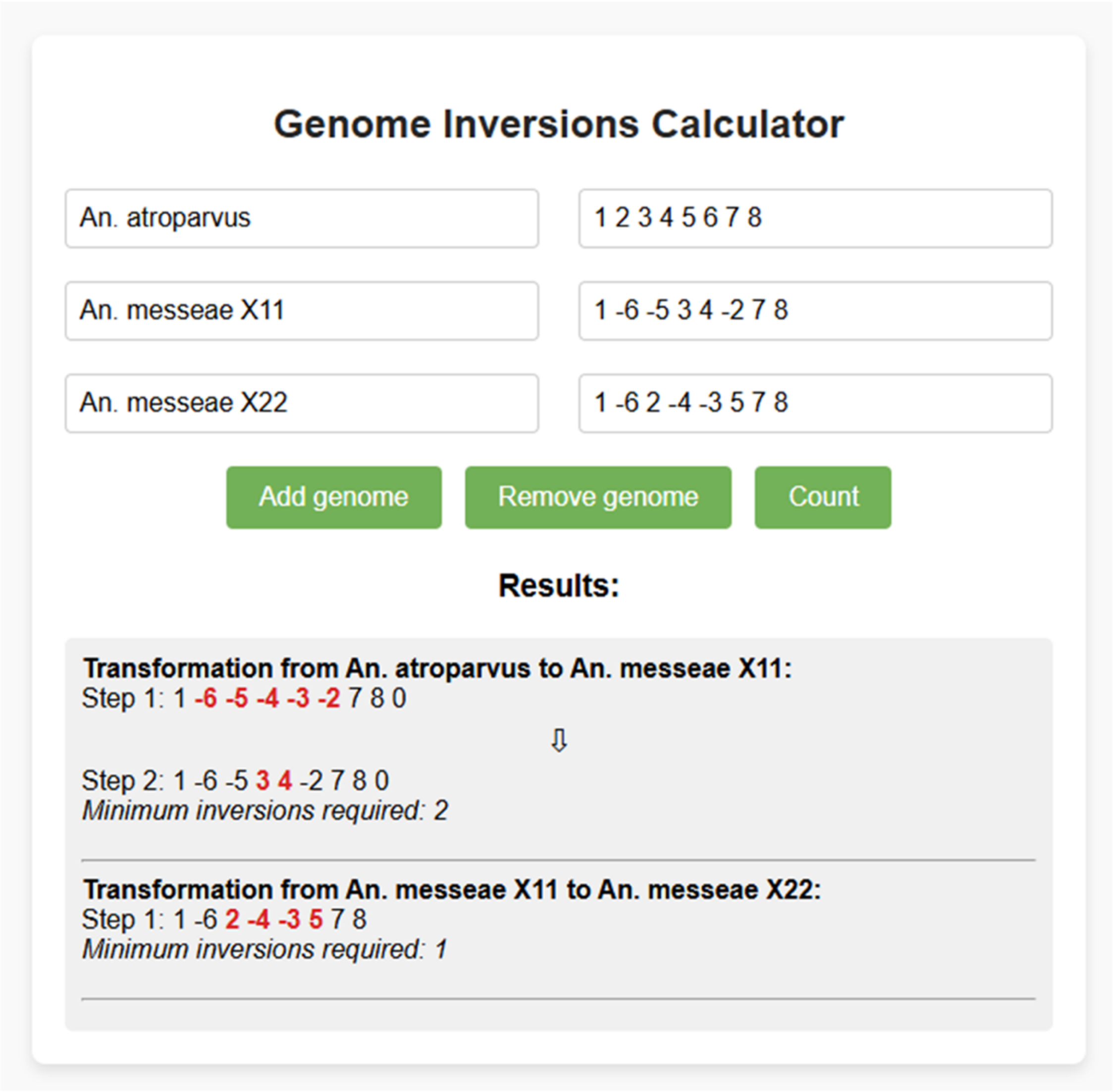

Figure S1. Illustration of the GIC tool in operation.

Table S1. Table S1. *An. atroparvus* marker genes used to map breakpoints of X chromosome polymorphic rearrangements in *An. messeae* and *An. daciae* using the AatrE3 assembly.

№	Gene ID	Primer sequencing (5' - 3')	Genome coordinates		Chromosomal region	
			start	end	***An. messeae* X1**	***An. messeae/daciae* X2/X0**
DNA probes for An. messeae X2						
1	AATE020937	ttttcttgcgcgaagctgac<br>gaacaggcagcacaaaccag	13,164,49	13,186,382(+)	1C	4B
2	AATE005475	cccgtcctttttctcctaca<br>ttgattttctgtcgtgtccg	13,236,449	13,246,292(-)	1C	4B
DNA probes for An. daciae X0						
3	AATE021012	ctgaatgggttcgactgt<br>gagtgacgctatcggaaa	4,398,009	4,404,032(-)	2A	2A
4	AATE006112	ctcctcaagaactgcctaac<br>tcagttttccgttgactcg	4,410,821	4,414,953(+)	2A	failed to map
5	AATE013223	gagaagcgaaggacgaga<br>ctcgcggtagagcctataa	4,528,886	4,534,883(+)	2A	transferred in 3R arm
6	AATE009672	gatcgacatggtgctgtc<br>gtcaccttcctcgtctgt	4,539,552	4,546,364(+)	2A	failed to map
7	AATE013154	actgagaacgcgaacatga<br>tatatggcgcaaggagtcg	4,571,860	4,577,721(-)	2A	5B
8	AATE000878	ctcgagcaaccttagcagg<br>gctgcagctcgtgtagaat	4,584,281	4,586,634(+)	2A	5B
9	AATE006100	agattacacgttggcatcc<br>cttaatagtgccagccgtt	4,594,775	4,596,266(+)	2A	5B
10	AATE012020	acattctgcatctcgcacag<br>catcacgtcggggaagtagt	4,602,182	4,608,814(-)	2A	5B
11	AATE011508	gtcgtgatgagagtcaatgg<br>cgatgttgtttgcttacgtg	4,609,127	4,610,269(-)	2A	5B
12	AATE014810	gttcacggaagactcacaaa<br>tcaatatcctctcgaagctga	4,611,080	4,611,919(+)	2A	5B
13	AATE000727	ggagtacgtctcgtcgg<br>tgcgtacctacctgacac	4,728,774	4,729,577(+)	2A	5B
14	AATE010388	gagttggctgtctaagaagg<br>ctgaatagtcccttcactgc	4,804,797	4,811,215(-)	2A	5B
15	AATE009337	aatccaccacctacgctaaa<br>ctagcccgatgaacgaaaag	4,874,801	4,878,654(-)	2A	5B
16	AATE020637	tttcctaagggcccagtttg<br>tcaacctgaactcgcacc	4,986,904	4,993,021(+)	2A	5B
17	AATE011314	caaccgtacagtctgaaacc	5,022,538	5,034,112(+)	2A	5B

		tcatttcaatcggctcatcg				
18	AATE020602	caagaagatcaccgagcac<br>gtgcgatcgagtacttcac	5,125,625	5,130,866(+)	2A	5B
19	AATE004697	tgctagaactacagtcaagt<br>ccaactatcataagcgagtg	17,057,247	17,066,579(-)	5B	2A
20	AATE003340	gcggttatcttttcctgttg<br>gtcgaagccgatgtagatta	17,252,047	17,256,383(+)	5A	2A
21	AATE018776	ttcccgtggacagaatctac<br>ccttcaccttatttcgctcg	17,260,494	17,265,377(-)	5A	failed to map
22	AATE010837	ataaagcatctgaggacgag<br>tttgcttgttcgatcttgag	17,409,992	17,412,127(+)	5A	transferred in 3L arm

Table S2. Characteristics of syntenic blocks (SBs) based on the AatrE3 assembly.

SB	Length of SB, bp	Number of genes	Average gene density per 100 Kb
1	1,458,895	122	8.36
2	526,550	42	7.97
3	2,401,223	179	7.45
4	7,775,450	533	7.11
5	902,442	53	5.87
6	1,272,996	86	6.76
7	2,733,670	193	7.06
8	no data		
